# Impact of Zero-Valent Iron Nanoparticles and Ampicillin on Adenosine Triphosphate and Lactate Metabolism in the Cyanobacterium *Fremyella diplosiphon*

**DOI:** 10.3390/microorganisms12030612

**Published:** 2024-03-19

**Authors:** Yavuz S. Yalcin, Busra N. Aydin, Viji Sitther

**Affiliations:** Department of Biology, Morgan State University, 1700 E. Cold Spring Lane, Baltimore, MD 21251, USA

**Keywords:** antibiotic, ATP, inter-thylakoid distance, lactate, nZVIs

## Abstract

In cyanobacteria, the interplay of ATP and lactate dynamics underpins cellular energetics; their pronounced shifts in response to zero-valent iron (nZVI) nanoparticles and ampicillin highlight the nuanced metabolic adaptations to environmental challenges. In this study, we investigated the impact of nZVIs and ampicillin on *Fremyella diplosiphon* cellular energetics as determined by adenosine triphosphate (ATP) content, intracellular and extracellular lactate levels, and their impact on cell morphology as visualized by transmission electron microscopy. While a significant increase in ATP concentration was observed in 0.8 mg/L ampicillin-treated cells compared to the untreated control, a significant decline was noted in cells treated with 3.2 mg/L nZVIs. ATP levels in the combination regimen of 0.8 mg/L ampicillin and 3.2 mg/L nZVIs were significantly elevated (*p* < 0.05) compared to the 3.2 mg/L nZVI treatment. Intracellular and extracellular lactate levels were significantly higher in 0.8 mg/L ampicillin, 3.2 mg/L nZVIs, and the combination regimen compared to the untreated control; however, extracellular lactate levels were the highest in cells treated with 3.2 mg/L nZVIs. Visualization of morphological changes indicated increased thylakoid membrane stacks and inter-thylakoidal distances in 3.2 mg/L nZVI-treated cells. Our findings demonstrate a complex interplay of nanoparticle and antibiotic-induced responses, highlighting the differential impact of these stressors on *F. diplosiphon* metabolism and cellular integrity.

## 1. Introduction

The complex interactions between environmental stressors such as nanoparticles and antibiotics have sparked renewed interest in understanding corresponding cellular responses in organisms. Unraveling the interdependent relationship among these external factors reveals the extraordinary capacity of photosynthetic microorganisms, particularly cyanobacteria, to adapt to multifaceted stressors [1]. As versatile microorganisms, cyanobacteria are valuable model organisms for investigating the impact of environmental stressors on metabolic and cellular processes [2]. These organisms, known for their role in renewable energy, have been identified as ideal for understanding microbial lipid synthesis due to their unique metabolic and physiological characteristics. Given their unique photomorphogenic adaptations and photo-regulatory mechanisms, cyanobacteria are capable of optimizing their photosynthetic efficiency in response to varying light conditions, an attribute that directly influences lipid production [3]. In addition, these organisms are recognized as carbon-neutral energy producers due to their use of carbon molecules they produce [4]. In an effort to combat global warming, the entire world community has set the difficult goal of reaching carbon neutrality by 2050, emphasizing the reduction of CO_2_ emissions through innovative strategies like the adoption of alternative energy sources and investment in green projects [5,6]. With their inherent capacity for photosynthetic CO_2_ capture, cyanobacteria play a pivotal role in these carbon neutrality strategies. These microbes offer a sustainable avenue for reducing atmospheric CO_2_ levels, leveraging their fast growth rates and wide metabolic potential to convert CO_2_ into useful biomass, thereby contributing to the development of bioenergy and bioproducts that support carbon-neutral objectives [7]. Moreover, a recent review by Sitther et al. [8] highlights the potential of cyanobacteria not only as a renewable energy source but also their significant role in industrial biotechnology, particularly in generating value-added bioproducts, bioplastics, and pharmaceuticals.

Researchers are increasingly interested in understanding the intracellular processes leading to macromolecule synthesis in cyanobacteria, including ATP, which provides energy storage and lactate, an important metabolite involved in the ATP-producing process. As an essential molecule for maintaining cellular vitality, ATP is necessary for enzymatic reactions in lipid synthesis, glycolysis, and the conversion of macromolecules in microorganisms [9]. Thus, the efficient utilization of ATP is crucial for the adequate functioning of these microbial entities. In addition to its role in cellular biosynthesis, ATP is also required for other energy-dependent processes, such as carbon fixation through photosynthesis and nitrogen fixation in cyanobacteria. Thus, various factors needed for ATP synthesis in enzymatic reactions have a substantial impact on the production of macromolecules such as lipids, proteins, and glucose [10]. As a result, a comprehensive understanding of the impact of environmental and cellular factors on cyanobacterial ATP levels is critical for optimizing lipid production, resulting in higher lipid yields [11].

Lactate, the final product of glycolysis generated by the lactate dehydrogenase (LDH) enzyme, is a crucial molecule in microbial lipid synthesis [12,13]. Lactate is also known as a biomarker of cellular integrity and membrane and is influenced by external stressors. With changes in the cyanobacterial cell membrane, lactate dynamically moves out of the cell, resulting in its accumulation in extracellular spaces [14]. Thus, elucidating lactate levels when cultures are exposed to stressors can provide comprehensive insight into membrane-related changes and cellular energy capacity [15].

Given that cyanobacteria play a crucial role in producing valuable bioproducts, it is unquestionably advantageous to comprehend how these organisms adapt to nZVI nanoparticles and antibiotics at optimal concentrations. Beyond conventional stressors such as salinity, light, cold, and heat shock, this study employs nZVIs and ampicillin to leverage their unique influences on cyanobacterial processes. Nanoscale zero-valent iron (nZVIs) molecules as iron-rich nanoparticles are an essential micronutrient for cyanobacteria and are required for fundamental cellular functions such as photosynthesis and respiration [16]. Furthermore, iron molecules can induce various reactive oxygen species (ROS) via Fenton-type reactions [17], which position nZVIs in a unique place both through mediation of biosynthesis and in terms of stress-related cellular processes. In addition to this, ampicillin, as a β-lactam antibiotic, is known to target bacterial cell membranes and enhance its permeability under specific conditions [18]. However, the comprehensive influence of ampicillin on various cyanobacterial species has not yet been fully understood. Additionally, different cyanobacterial species exhibit varying levels of susceptibility to this antibiotic [19,20]. Our previous report on *F. diplosiphon* B481-SD treated with ampicillin at concentrations ranging from 0.2 mg/L to 25.6 mg/L revealed significantly enhanced phycocyanin and chlorophyll *a* autofluorescence when compared to the untreated control [21].

In light of this, the current study aimed to investigate the effects of ampicillin and nZVIs, and their combined effect on the bioenergetics and stress responses of *Fremyella diplosiphon* (Bornet & Flahault) Drouet (currently regarded as *Microchaete diplosiphon* Gomont ex Bornet & Flahault), a model cyanobacterium known for its ability to absorb different wavelengths of light. In addition, TEM was used to visualize intracellular changes in *F. diplosiphon* cell structure and integrity, further complementing biochemical analyses and providing insight into the physical and morphological alterations triggered by the treatments.

## 2. Materials and Methods

### 2.1. Cyanobacterial Strain, Growth Conditions, and Treatments

*F. diplosiphon* strain B481-SD, which was overexpressed with the sterol desaturase gene for enhanced lipid production (accession MH329183), was used in the study. The organism was cultivated in BG-11/HEPES medium, under wide-spectrum red light (650 nm) at a light fluence rate of 30 μmol/m^2^/s, using the model LI-190SA quantum sensor (Li-Cor, Lincoln, NE, USA). These conditions were facilitated by an Innova 44R shaker (Eppendorf, Hamburg, Germany), which provided continuous agitation at 170 rpm and an incubation temperature of 28 °C. Iron nZVIs were obtained from the Nano Iron Company (Rajhrad, Czechia), and ampicillin, a β-lactam group antibiotic (Sigma Aldrich, St. Louis, MO, USA), was used in this study. An optimal concentration of 0.8 mg/L ampicillin and 3.2 mg/L nZVIs were selected based on prior reports in *F. diplosiphon* [21,22].

*F. diplosiphon* B481-SD liquid cultures were grown under the conditions mentioned above to achieve an optical density of 0.6 at 750 nm and subjected to the following treatments: ampicillin at 0.8 mg/L, nZVIs at 3.2 mg/L, and a combined regimen incorporating both ampicillin at 0.8 mg/L and nZVIs at 3.2 mg/L. Control cultures were grown in the absence of any treatment with antibiotics or nZVIs. The entire experiment was conducted in triplicate and repeated once for validation.

### 2.2. ATP Extraction and Measurement

B481-SD cultures grown under the conditions mentioned in Section 2.1 were harvested on day 12 (0.6 OD_750_) by centrifugation at 13,000 rpm for 3 min. The supernatant was discarded, and pellets were resuspended in 100 μL of 1% ice-cold trichloroacetic acid solution. Samples were vortexed for 30 s and centrifuged at 13,000 rpm for 10 min at 4 °C to collect the supernatant. Trichloroacetic acid solution was neutralized by adding 100 μL of 1 M Tris-Acetate buffer (pH 7.8), and the ATP-containing solution was diluted to 1 mL in deionized water. ATP content was measured using the ATP determination kit (Thermo Fisher Scientific, Molecular Probes Inc., Waltham, MA, USA), according to the manufacturer’s instructions. Briefly, 10 μL of the diluted ATP solution was mixed with the ATP detection reagent, and the luminescence signal was measured using a Synergy H1 Multimode microplate reader equipped with Gen5 software version 2.10 (Bio Tek, Santa Clara, CA, USA). ATP concentration was calculated using a standard curve prepared in conjunction with the sample measurement.

### 2.3. Extracellular and Intracellular L-Lactate Quantification

L-Lactate, a metabolic byproduct of glycolysis, known for its significant role as an energy provider in cells, was quantified via fluorometric assay. To perform the assay, 1 mL of culture from each treatment was centrifuged at 4000× *g* for 6 min at 4 °C, and the supernatant was preserved at −20 °C. Sample analysis was conducted using the L-Lactate Assay kit (Cayman Chemical, Ann Arbor, MI, USA), according to the manufacturer’s instructions. The lactate concentrations in the supernatant were corrected using the lactate concentrations in the blanks. Measurements were made on 96-well clear plates (Corning Inc., Corning, NY, USA), using a Synergy H1 Multimode microplate reader as mentioned in Section 2.2.

### 2.4. Transmission Electron Microscopy

Cultures grown under the conditions mentioned in Section 2.1 were centrifuged at 4000 rpm for 10 min and fixed with glutaraldehyde to a final concentration of 2.5% for 5 min. The samples were washed thrice with 5 mM HEPES buffer (pH 8.0) and subsequently post-fixed overnight at 4 °C with a 2% solution of potassium permanganate. The cells were rinsed between 8 and 10 times with deionized water and suspended in a 2% solution of SeaKem agarose (FMC Bioproducts, Rockland, ME, USA). Upon solidification, the agarose-embedded samples were sectioned into ~2 mm cubes and dehydrated through a series of ethanol baths, incrementally increasing in concentration from 70% to 95% twice for 10 min each, followed by three 20 min immersions in 100% ethanol.

The samples were subsequently prepared for resin infiltration by incubation in propylene oxide for a quarter of an hour. The first stage of resin infiltration was performed in a 1:2 mixture of propylene oxide and Epon for 30 min, followed by a 1:3 mixture for 90 min at room temperature. The agarose cubes were embedded in pure Epon and left to polymerize for 24 h at 40 °C, followed by an additional 48 h at 60 °C. The polymerized samples were ultrathin sectioned and examined in a Philips Tecnai 10 Transmission Electron Microscope (Phillips, Amsterdam, The Netherlands) at 80 kV. Micrographs were taken with a two-second exposure time on medium format film and subsequently digitized using a transmitted light scanner. This procedure was performed at the Johns Hopkins University microscope facility (Baltimore, MD, USA).

### 2.5. Statistical Analysis

All data were analyzed as the mean ± standard deviation of three independent replicates. Statistical analysis was performed using one-way analysis of variance, followed by Tukey’s post hoc test, with *p* < 0.05 considered to be statistically significant. SPSS 28.0 (IBM Corporation, Armonk, NY, USA) was used to analyze and plot the data.

## 3. Results and Discussion

As a cornerstone of cellular energetics, ATP is a quintessential energy carrier that serves as an informative indicator of shifts in metabolic activity and organismal stress responses, reflecting the dynamic balance between energy production and consumption. [23,24]. In this study, we investigated the effects of nZVIs and ampicillin on the bioenergetics of *F. diplosiphon* by understanding how these environmental stressors impact ATP levels, a key determinant of metabolic activity. The untreated control served as a baseline ATP concentration and reference point for comparative analysis.

### 3.1. Assessment of ATP Levels in F. diplosiphon under Ampicillin and nZVIs’ Treatments

**Figure 1:**
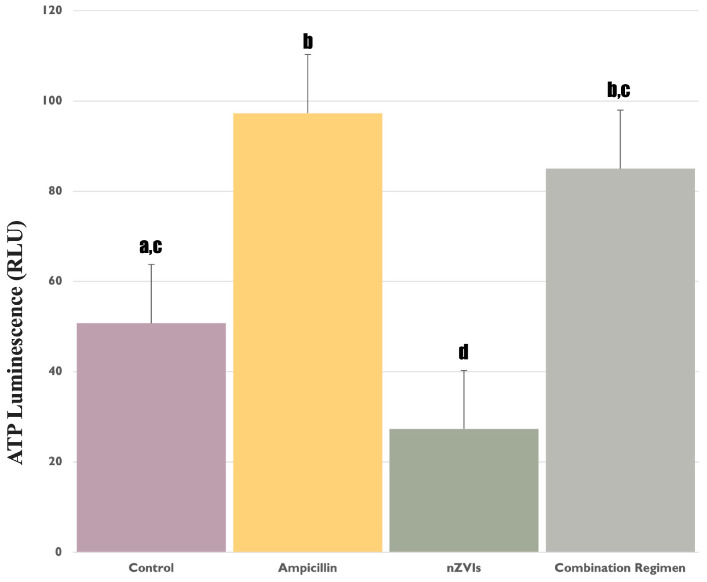
ATP levels in *Fremyella diplosiphon* treated with 0.8 mg/L ampicillin, 3.2 mg/L nZVIs, combination regimen (0.8 mg/L ampicillin and 3.2 mg/L nZVIs), and untreated control measured using a luciferase-based assay. Error bars annotated with different letters (e.g., d and b, c) denote significant differences (*p* < 0.05), while similar letters (e.g., a, c and b, c) indicate no significant differences (*p* > 0.05) among the groups as determined by Tukey’s post-hoc test. Error bars represent the standard error (SE) of the mean.

We observed a significant increase (*p* < 0.05) in ATP concentration of the strain to 0.8 mg/L ampicillin, compared to the untreated control (Figure 1), indicating a response consistent with our earlier report in which ampicillin enhanced oxidative stress [14]. In light of this, it might be reasoned that the enhanced ATP synthesis is an outcome of the organism ramping up cellular activity in an effort to mitigate the potentially deleterious effects of the antibiotic. In addition, a significant decrease (*p* < 0.05) in ATP levels was observed in B481-SD treated with 3.2 mg/L nZVIs compared to 0.8 mg/L ampicillin and the combination regimen (Figure 1); this level was also significantly lower (*p* < 0.05) than the untreated control, which established the baseline ATP levels. We hypothesize that the stress could potentially impair the cellular machinery tasked with ATP synthesis, resulting in a significant decline in ATP yield. In a study by Gichuki et al. [25], significant increases in dichlorofluorescein and malondialdehyde content, indicating oxidative stress in *F. diplosiphon* B481-SD subjected to nZVIs exceeding 3.2 mg/L, were reported. Another possible explanation could be attributed to the interference of nZVIs with the electron transport chain that is integral to photosynthesis, subsequently hampering ATP production [26].

Interestingly, the scenario unfolded differently when the organism was subjected to the combination regimen of 0.8 mg/L ampicillin and 3.2 mg/L nZVIs. While ATP levels were significantly elevated (*p* < 0.05) compared to 3.2 mg/L nZVIs, it was not significantly different (*p* > 0.05) than 0.8 mg/L ampicillin and the untreated control (Figure 1). This suggests an intriguing interplay between the stress responses elicited by the antibiotic and nZVIs. It is possible that ampicillin could have augmented ATP activity in the strain due to the fact that it is the common treatment in both regimens, reflecting heightened cellular activity and possibly triggering ATP-dependent cellular protection systems. Moreover, the substantial differences observed in the various treatments indicate that ampicillin could be linked to its innate ability to impact cell wall synthesis. This effect could have stemmed from increased membrane permeability, facilitating the shift of nZVIs into the intracellular space, therefore mitigating potential detrimental effects that may arise from the accumulation of nZVIs around the cell. This observation is consistent with a study by Jiang et al. [27], in which multifaceted stressors, including antibiotics, were reported to enhance cellular growth and functionality in *Microcystis aeruginosa* (Kützing) Kützing in accordance with the hormesis phenomenon.

### 3.2. Impact of Ampicillin and nZVIs’ Treatments on Lactate Levels

#### 3.2.1. Extracellular Lactate Levels

**Figure 2:**
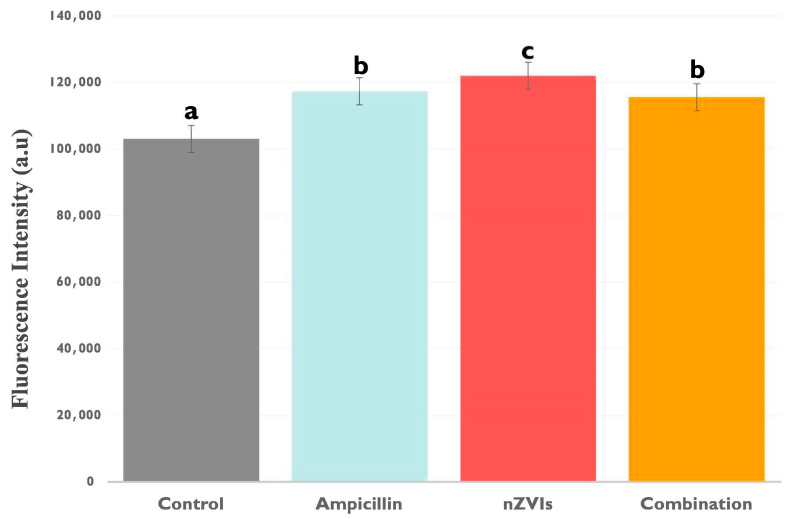
Extracellular lactate levels in B481-SD treated with 0.8 mg/L ampicillin, 3.2 mg/L nZVIs, the combination regimen (0.8 mg/L ampicillin and 3.2 mg/L nZVIs), and untreated control. Lactate levels were measured using enzymatic assay methods. Error bars annotated with different letters denote significant differences (*p* < 0.05), while similar letters indicate no significant differences (*p* > 0.05) among the groups as determined by Tukey’s post-hoc test. Error bars represent the standard error (SE) of the mean.

Extracellular lactate levels, which are known to serve as a valuable marker for cellular stress and membrane integrity under diverse environmental conditions, served as an indicator of *F. diplosiphon* exposed to nZVIs and ampicillin. Interestingly, B481-SD grown in 0.8 mg/L ampicillin, 3.2 mg/L nZVIs, and the combination regimen resulted in significantly higher (*p <* 0.05) extracellular lactate levels compared to the untreated control (Figure 2). It is possible that the increase in cellular stress to external chemicals could have enhanced cellular damage and subsequent lactate release, although the severity of this damage did not reach a level that caused cell death or a decrease in autofluorescence. Notably, extracellular lactate levels in 3.2 mg/L nZVI-treated cells were significantly higher (*p* < 0.05) than B481-SD treated with 0.8 mg/L ampicillin and the combination regimen (Figure 2). Since the release of lactate into the extracellular environment is well-documented as a marker of cellular distress [28,29], this observation is likely caused by the potent oxidative stress of nZVIs, leading to considerable cellular damage and the release of intracellular components such as lactate. Owing to the small size and high reactivity of nZVIs [30], these nanoparticles might accumulate rapidly around the cell, impacting the cellular membrane, which subsequently could have contributed to the observed increase in extracellular lactate. These findings are supported by a study that demonstrated visible FeO nanoparticle accumulation around the cell membrane border and increased oxidative stress in cyanobacteria [26], further substantiating the impact of iron nanoparticles on the cell membrane and the potential increase in extracellular lactate levels. In response to membrane thinning and enhanced permeability associated with ampicillin, the harmful effects of accumulated nanoparticles around the cell may be mitigated by the leakage of nZVIs into the cells [31]. Moreover, it is possible that increased intracellular iron could potentially be used as cofactors for reactions that enhance the synthesis of macromolecules such as lipids and glucose, thereby improving biofuel production efficiency and overall metabolic robustness.

On the other hand, augmented extracellular lactate levels in 0.8 mg/L ampicillin-treated B481-SD compared to the untreated control could be attributed to its stress response to the antibiotic. By inhibiting the synthesis of cell wall peptidoglycan, it is possible that ampicillin could have induced cellular instability and damage, resulting in the release of LDH into the extracellular space. However, it is crucial to note that both 0.8 mg/L ampicillin and the combination regimen (0.8 mg/L ampicillin + 3.2 mg/L nZVIs) enhanced extracellular lactate levels due to the possible cellular stress response. In our previous study, we reported improved cellular growth and pigmentation at the same concentrations with B481-SD, possibly due to a hormetic effect [21]. Based on our current findings, we hypothesize that this growth response may be balanced by intracellular protection systems such as antioxidant enzymes or cellular efflux channels that mitigate the cellular stress induced by these chemicals [32]. In addition, the significant increase in (*p* < 0.05) extracellular lactate levels observed in B481-SD treated with ampicillin, nZVIs, and the combination regimen (Figure 2) may be attributed to a shift toward anaerobic metabolism, forcing the cells to rely on glycolysis, where lactate is produced.

Moreover, similar ATP and extracellular lactate levels in B481-SD treated with 0.8 mg/L ampicillin and the combination regimen could be caused by increased membrane permeability. In normal conditions, ATP is contained within cells due to its large size and negative charge, which prevents this essential molecule from leaking into the extracellular space [33]. However, damage to the cell membrane or enhanced membrane permeability can cause ATP, lactate, and similar intracellular products to leak out easily and increase their extracellular levels [34]. Our previous results support the findings that ampicillin and the combination regimens could result in significant changes to the cell wall of the cultures. Specifically, in B481-SD treated with 0.8 mg/L ampicillin, 3.2 mg/L nZVIs, the combination regimen and the untreated control, cell wall thinning was measured at 11.3 nm, 13 nm, 8 nm, and 14 nm, respectively [31].

#### 3.2.2. Intracellular Lactate Levels

In this study, intracellular lactate levels were evaluated to gain insights into the intracellular metabolic dynamics of *F. diplosiphon* under ampicillin and nZVI-mediated stress.

**Figure 3:**
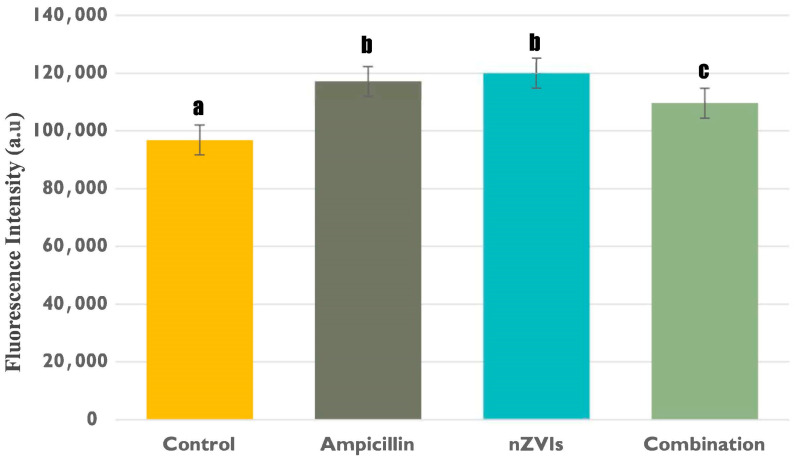
Intracellular lactate levels in B481-SD treated with 0.8 mg/L ampicillin, 3.2 mg/L nZVIs, the combination regimen (0.8 mg/L ampicillin and 3.2 mg/L nZVIs), and untreated control. Lactate levels were measured using a colorimetric LDH assay. Error bars annotated with different letters denote significant differences (*p* < 0.05), while similar letters indicate no significant differences (*p* > 0.05) among the groups as determined by Tukey’s post-hoc test. Error bars represent the standard error (SE) of the mean.

Intracellular lactate levels in B481-SD treated with 0.8 mg/L ampicillin, 3.2 mg/L nZVIs, and the combination regimen were observed to be significantly higher compared to the untreated control (Figure 3); however, the highest intracellular lactate levels were measured in individual treatments of 3.2 mg/L nZVIs and 0.8 mg/L ampicillin. Therefore, increased intracellular lactate levels may indicate a substantial metabolic response in B481-SD treated with 3.2 mg/L nZVI and 0.8 mg/L ampicillin, possibly resulting in increased lactate production or retention within the cells. The significant alteration of metabolic pathways within *F. diplosiphon* cells in response to antibiotic and nanoparticle stressors can be underscored by variations in intracellular lactate levels [26]. Although the combination regimen included both chemicals, intracellular lactate levels were significantly lower than individual treatments. It can be reasoned that increased Monounsaturated Fatty Acids (MUFAs), which are known to reduce oxidative stress [35] and increase cell membrane fluidity [36], can mitigate the detrimental effects and support cell survival. Similarly, a study by Yalcin et al. [31] reported that the combination regimen can produce MUFAs in 2–3-fold. Thus, we perceive that the combined stressors can instigate more complex cellular responses, resulting in different lactate levels compared to individual treatments and untreated controls.

A comparison of intracellular and extracellular lactate levels (Figure 2 and Figure 3) in B481-SD treated with ampicillin and 3.2 mg/L nZVIs showed statistically higher extracellular lactate levels; however, intracellular lactate levels between the treatments were not significantly different (*p* < 0.05). The dichotomy we observed between intracellular and extracellular lactate levels might be derived from the different roles and regulatory mechanisms of this product within and outside the cell [37]. As intracellular lactate primarily contributes to metabolic processes such as catalyzing the conversion of pyruvate to lactate during glycolysis and supporting ATP production [19], individual treatment of nZVIs and ampicillin could affect intracellular metabolic activity and energy metabolism. Conversely, extracellular lactate is primarily a biomarker for cell membrane integrity, as it is released into the extracellular space following damage to the cell membrane or cell death [17]. Therefore, the observed increase in extracellular lactate levels in B481-SD treated with nZVIs may be attributed to concentration-related changes in the cell wall and membrane. According to a study by Kumar et al. [38], when the cyanobacterium type *Nostoc ellipsosporum* (Rabenhorst ex Bornet et Flahault) was treated with FeO nanoparticles at concentrations ranging from 10 to 100 mg/L, an increase in LDH levels consistent with the escalating concentrations was reported, suggesting a dose-dependent response to the nanoparticle treatment.

### 3.3. Microscopic Analysis

#### ATP Dynamics and Cellular Morphology of *F. diplosiphon* in Response to nZVI and Ampicillin Treatment

We employed TEM analysis to uncover correlations between cellular ATP levels and distinct morphological changes in each treatment. In this regard, we focused on examining the intracellular ATP concentrations and their correlation with any concurrent cellular and thylakoid membrane changes in *F. diplosiphon* under the influence of nZVIs, ampicillin, and their combined application.

**Figure 4:**
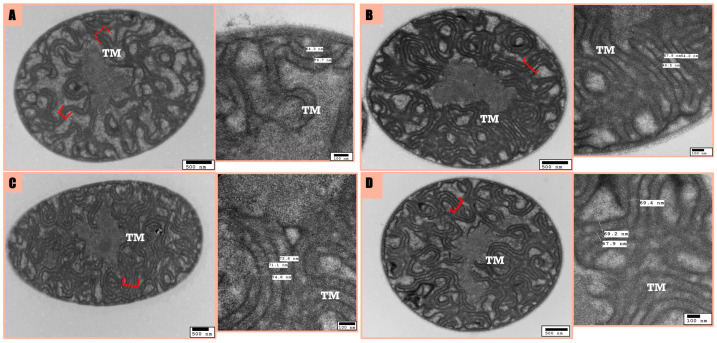
Transmission Electron Microscopy (TEM) observations of inter-thylakoidal distances in *Fremyella diplosiphon* B481-SD treated with untreated control (**A**), 0.8 mg/L ampicillin (**B**), 3.2 mg/L nZVIs (**C**), and a combination treatment of 0.8 mg/L ampicillin and 3.2 mg/L nZVIs (**D**). The stack of thylakoid membranes is indicated in red parentheses. TM: Thylakoid membrane.

B481-SD treated with 3.2 mg/L nZVIs resulted in a decrease in ATP, which was accompanied by changes in thylakoid membranes and an increase in inter-thylakoidal distance compared to the untreated control and 0.8 mg/L ampicillin. This concurs with the fact that ATP depletion may drive morphological adaptations as part of the cellular stress response. However, increased thylakoid membrane counts in the B481-SD treated with 0.8 mg/L ampicillin can explain the increase in ATP concentrations. ATP changes indicated that disrupted internal organelles might reflect a defensive response to treatments, suggesting that ATP dynamics influence the extent of these morphological responses. The potential for an increased number or volume of thylakoids in the ampicillin, nZVIs, and the combination regimen compared to the untreated control (Figure 4) might account for stable ATP levels, as thylakoid membranes are pivotal for ATP synthesis in cyanobacteria.

In the combination regimen and ampicillin treatments, the mean inter-thylakoidal distance among thylakoid membranes was measured at 68.8 nm and 53.86 nm, respectively; however, it was 72 and 72.8 nm for the untreated control and nZVIs (Figure 4). Therefore, the inter-thylakoidal distance might be crucial for ATP synthesis, as shorter distances can result in increased proton gradient, leading to augmented ATP production. Similarly, the ability to alter inter-thylakoidal distances was observed in a study by Stingacui et al. [39], where *Synechocystis* sp. PCC 6803 demonstrated a 3–4-fold increase in mobility under dark conditions, thus enhancing photosynthesis compared to light conditions. Therefore, cyanobacterial cells can acclimate to variable environmental changes by modifying thylakoid membranes, which are vital photosynthetic structures, thereby enhancing their resilience. On the other hand, the size of the pigment positioned within the inter-thylakoidal membrane is crucial, as shown by Liberton et al. [40], where a minimal distance based on the size of phycobiliproteins of 4 nm should be provided for the cultures to carry out photosynthesis. Furthermore, we observed this minimal distance in all the treatments tested in this study.

In summary, our findings underscore the intimate link between cellular energy dynamics and morphological alterations of the strain in response to external stressors. Future research is warranted to dissect the molecular mechanisms underlying these observations and will offer valuable insights into *F. diplosiphon* cellular resilience and survival tactics under varied stress conditions. We observed disorganized thylakoid membranes and increased intracellular space in TEM studies, indicating a possible cellular stress response to nZVI exposure. This supports the notion that cellular morphology and energy dynamics are closely intertwined, with changes in ATP levels potentially contributing to the morphological adjustments mentioned above. Our findings underline the substantial role of ATP concentration in dictating cellular responses to external stressors, reflected through morphological adaptations.

## 4. Conclusions

Our study reveals a fascinating interplay between cellular ATP and lactate levels, morphological changes, and environmental stressors in the cyanobacterium *F. diplosiphon*. We observed that changes in ATP levels, driven by the effects of nZVIs and ampicillin, correlate to alterations in thylakoid membranes and inter-thylakoidal distances, thus impacting the photosynthetic capability of cyanobacterium. These findings provide insights into the cellular resilience of the strain, highlighting its ability to acclimate to varying environmental conditions by altering vital photosynthetic structures. Our study underscores the importance of ATP dynamics in understanding cellular responses to external stressors, which are significant for environmental and microbial studies. Critical insights into cyanobacterial adaptive mechanisms under ampicillin and nZVI-stress advances our understanding of environmental stress biology. This knowledge not only facilitates the efficient harnessing of the potential of *F. diplosiphon* but also paves the way for sustainable and innovative applications across various industries. By delving into the intricacies of *F. diplosiphon* chemical adaptations, we can unlock its full potential in enhancing value-added bioproducts and biofuel generation for economic viability. Future research to explore the genetic underpinnings of these adaptive responses will offer avenues for genetic engineering to optimize cyanobacterial strains for industrial use. This direction not only supports the sustainable production of biofuels and bioproducts but also contributes to global efforts in carbon capture and sequestration, aligning with the objectives of carbon neutrality and a greener economy.

## Data Availability

Data are contained within the article.
